# Association between grip strength and electrical properties measured by bioimpedance spectroscopy in women with dementia aged 77 to 97 years living in group homes

**DOI:** 10.3892/mi.2024.157

**Published:** 2024-04-19

**Authors:** Kenichiro Yasutake, Hideaki Kumahara, Keisuke Shiose, Marina Kawano, Ryoma Michishita

**Affiliations:** 1 Department of Nutritional Sciences, Faculty of Nutritional Sciences, Nakamura Gakuen University, Fukuoka 814-0198, Japan; 2 Graduate School of Nutritional Sciences, Nakamura Gakuen University, Fukuoka 814-0198, Japan; 3 Faculty of Education, University of Miyazaki, Miyazaki 889-2192, Japan; 4 Faculty of Sports and Health Science, Fukuoka University, Fukuoka 814-0180, Japan

**Keywords:** muscle quality, body composition, nutritional assessment, muscle strength, electric impedance

## Abstract

Electrical properties estimated from the electrical resistance of the human body can serve as indicators of muscle tissue status and the risk of developing sarcopenia; however, to date, at least to the best of our knowledge, no studies have performed such an assessment in older individuals with advanced dementia. The present study examined the associations between grip strength, body composition and electrical properties using bioimpedance spectroscopy (BIS) in women aged 77-97 years residing in dementia group homes. A total of 33 participants were enrolled with an average age of 88.1±5.2 years; 57.6% of the participants had moderate or severe dementia. The resistance values of the participants were measured in the whole body, upper limbs and lower limbs using BIS, and their body composition, muscle mass index and electrical properties were estimated as indicators of muscle quality. In addition, grip strength was measured and the participants were classified into three groups (high, low and non-measurable) according to their cognitive function. The effect size (partial eta-squared and Cohen's d) was also evaluated. The Shapiro-Wilk test was used to assess the distribution of each variable; variables with non-normal distributions were analyzed following log transformation. Continuous variables were analyzed using a one-way analysis of variance and the Tukey-Kramer post hoc test was used. The post hoc sample size (statistical power: 1-β) analysis revealed a power of ~80% (i.e., 76.1-88.7%), considering the minimum power for sufficient participants. No differences were found in body composition or muscle mass index among the three grip strength groups. As regards the upper limbs, the electrical properties of the characteristic frequencies were significant (P=0.006; effect size, large), and the membrane capacitance (P=0.005; effect size, large) was significantly higher in the high-dose group than in the other groups. A significant association was detected among grip strength, upper limb characteristic frequency and membrane capacitance. Hence, electrical properties may be an indicator of muscle quality in older women identified as needing care for dementia.

## Introduction

The number of individuals with dementia worldwide is increasing steadily and is expected to reach 74.7 million by the year 2030(1). Similarly, the number of patients with dementia in Japan is rapidly increasing owing to the aging population (2). The prevention and treatment of dementia, as well as post-dementia life support, are currently among the most critical issues in public health. For example, in Japan, group homes in each community are positioned as an crucial base of a system to support individuals with dementia in society, with efforts being made to ensure that even those with advanced dementia identified as requiring a high nursing care level based on the Certification of Needed Long-Term Care, can continue to live in their own way in their familiar communities (3).

Maintaining muscle mass and strength associated with the basic activities of daily living (ADL) is of particular importance in patients with dementia (4). Previous research has suggested that decreased muscle mass and strength in patients with dementia may be associated with the pathophysiology and progression of dementia. Burns *et al* (5) reported that a decreasing lean mass is significantly associated with the atrophy of whole brain volume in early Alzheimer's disease (AD). Moreover, Moon *et al* (6) reported that the skeletal muscle mass index measured using dual-energy X-ray absorptiometry in patients with AD is a predictor of the progression of dementia. The same authors also found that muscle strength in patients with moderate-stage AD was associated with the volume ratio of the left hippocampus (7). Furthermore, in addition to muscle mass and strength, the concept of muscle quality has been proposed as an indicator of the state of muscle tissue (8). A previous systematic review demonstrated that the phase angle of muscle quality markers (9) measured using multifrequency bioelectrical impedance analysis in older individuals without dementia and in injured patients was a predictive indicator of sarcopenia. This indicates the importance of assessing muscle mass, strength and quality in order to prevent the onset of and delay the progression of sarcopenia (10).

Recently, bioimpedance spectroscopy (BIS) was developed as a relatively inexpensive and portable method for the non-invasive evaluation of body properties. BIS uses low- to high-frequency currents to distinguish between the resistance of intracellular components (Ri) and the resistance of extracellular components (Re) and is generally used to estimate body composition. In addition, BIS can estimate electrical properties, such as the characteristic frequency (f characteristic), membrane capacitance and phase angle from the resistance of the component (reactance, Xc; resistance, R) (9). These electrical properties indicate the performance of the cell membrane as a capacitor and are considered to be primarily related to the body water ratio (extracellular water/intracellular water) of muscle cells and the structural properties of muscle cells (11,12). Indeed, electrical properties are related to muscle strength and motor function in young and older individuals (13,14). However, to the best of our knowledge, there are no studies available to date on the associations between muscle strength, body composition and electrical properties, as measured using BIS, in older individuals with advanced dementia identified as requiring care. Furthermore, the potential usefulness of measuring electrical properties for assessing muscle strength and muscle quality need to be evaluated, as no valid estimation formula currently exists to assess body composition in frail elderly individuals using the impedance method.

The aim of the present study was to examine the association between grip strength, which reflects whole-body muscle strength (15,16), and body composition and electrical properties measured using BIS in older individuals with advanced dementia who reside in group homes. Collectively, investigating the association between muscle strength and electrical properties in patients with dementia may contribute to the discovery of novel indicators for the prevention of the onset and progression of the disease. This may also improve the quality of care required to maintain ADL through the visualization of muscle quality.

## Patients and methods

### Study participants. Recruiting participants

The contents of the research were explained to the staff members and the families of the residents' (proxies) at three group homes in Japan. At the recruitment stage, participants were not excluded based on etiology, sex, age, or medication status for dementia. The target population comprised 54 residents whose families agreed to participate in the study in April, 2019.

*Exclusion criteria.* Participants were excluded in the case that the nursing care staff could not control the symptoms of dementia or comorbidities of the patients, if they had been bedridden for >1 week, and in the event that the patients or their relatives (proxies) refused to participate before or during the investigation.

### Ethical considerations

The present study was approved by the Ethics Committee of Nakamura Gakuen University, Fukuoka, Japan (no. Rinri-17-004) and was performed in accordance with the Declaration of Helsinki. In addition to aspects of personal information protection, each facility director of the three group homes and the residents' families (proxies) were informed of the outline of the study, voluntary enrollment and withdrawal policies (participants were free to withdraw from participation without discrimination). They were also informed that the results would be made public at scientific conferences and in the literature at the Information Network Center (UMIN) (UMIN000036409) prior to the start of the study.

### Questionnaires

The data of the participants were collected from each questionnaire. Their sex, age, etiology of dementia and nursing care levels, based on the Certification of Needed Long-Term Care (17) were collected from their medical records. The nursing care level was divided based on severity as follows: Nursing care level 1, included individuals whose capacity to carry out instrumental activities of daily living was lower that of those who required support and partial nursing care; nursing care level 2, included individuals who, in addition to requiring nursing care level 1, also required partial nursing care related to ADL; nursing care level 3, included individuals whose capacity for daily living and instrumental activities was substantially lower than that of those in nursing care level 2, and who required nursing care in most aspects of life; nursing care level 4, included individuals who, in addition to having difficulties experienced by those in the nursing care level 3 category, had less mobility and more difficulty leading everyday life without nursing care; nursing care level 5, included individuals whose mobility was more constrained than those in level 4, and for whom it was relatively impossible to lead everyday life without nursing care (17) (http://www.arsvi.com/d/a02i-e.htm).

The Clinical Dementia Rating (CDR) (18) and the Barthel index (BI) for ADL (19) were determined based on assessments administered by the examiners and nursing care staff.

### Physical functional assessments. Measurement outline

All physical function assessments were performed by three examiners with the assistance of care staff. At this time, to avoid measurement errors between examiners, each measurement was performed by the same examiner. Generally, physical functional assessments measure the fasting status early in the morning; however, it was not possible to unify the measurement conditions related to the measurement times in the present study. In fact, measuring physical function required >30 min per individual due to the characteristics associated with advanced dementia and relatively high-level nursing care.

*Height and weight.* The height and weight of the participants were measured to the nearest 0.1 cm or 0.1 kg while wearing light clothing. To measure height, a one-step stature meter (Seca 217; seca GmbH & Co. KG) was used for those who were able to maintain their back muscles in an upright position. Alternatively, a measuring tape was used in the supine position on a bed for those who had difficulty standing. Weight was measured using a scale (BF-049-WH; TANITA) for those who could maintain a standing position and a barrier-free scale for wheelchairs (AD-6106NW; A&D Medical) for those who were confined to a wheelchair. BMI values were calculated from height and weight values.

*Grip strength.* Grip strength was measured using a Smedley-type digital grip force meter (Takei Electric Industries, Ltd.; Grip-D measuring range, 5.0-100.0 kg). The participants were directed to grasp with maximum effort while sitting on a chair with their elbows at right angles. The handgrip strength meter did not come into contact with the bodies of the participants while the examiner supported their elbows (20). In the case that the participants were unable to communicate fully due to cognitive dysfunction, the nursing care staff encouraged them to perform the measurements. The grip width was adjusted such that the second joint of the index finger was at a right angle. The measurements were performed twice on both the left and right hands (0.1 kgf; maximum kgf value was used as the representative value). The lower limit of the grip-strength meter measured values was 5 kgf; that is if the measurement was <5 kgf, it was recorded as 0 kgf. The criterion for determining those capable of demonstrating grip strength was based on the ability of the participants to exert themselves in a handshake position. If the examiners felt a degree of grip strength, the three examiners and nursing care staff agreed that the participants were able to measure their grip strength.

The participants were categorized into three groups based on their grip strength values as follows: i) The high group included those whose grip strength values exceeded the median value; ii) the low group included those whose grip strength values were lower than the median value, including those who held the meter, but had a recorded strength of 0 kgf; iii) the non-measurable group included those whose grip strength was non-measurable due to deficits in cognitive function.

**Figure 1:**
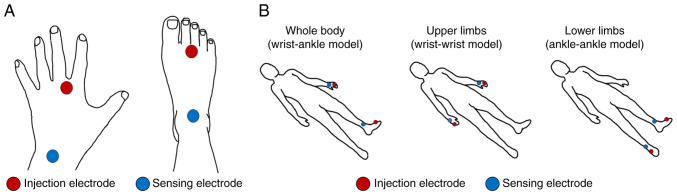
Attachment positions of the injection and sensing electrodes of the bioelectrical impedance spectrometer (bioimpedance spectroscopy method). (A) Injection electrodes (electrocardiographic electrodes 2330; 3M) were placed on both sides of the body and on the dorsal surface of both hands and feet, proximal to the metacarpophalangeal and metatarsophalangeal joints, respectively. Sensing electrodes were then placed on both sides of the body at the middle of an imaginary line on the dorsum of the wrist, joining the bony prominences of the radius and ulna, and at the middle of the anterior surface of the ankle, on an imaginary line joining the medial and lateral malleoli. (B) The whole body (wrist-ankle model) was measured alternately on the left and right sides; the upper limbs (wrist-ankle model) and the lower limbs (ankle-ankle model) were measured in this order.

*Measurement of body composition and estimation of electrical properties.* Body composition, muscle mass and electrical properties were measured using the BIS (SFB7; ImpediMed). Prior to performing the measurements, the presence of pacemakers and internal metal objects (e.g., bolts) was queried; the length (L: cm) of each body part (shoulder width, left and right upper and lower limbs) was measured using a measuring tape in 0.1-cm units. Shoulder width was measured as the length connecting the top of both shoulders, upper limb width as the length from the top of the shoulder to the wrist, and lower limb width from the greater trochanter to the ankles. Injection electrodes (electrocardiographic electrodes 2330; 3M) were placed on both sides of the body and on the dorsal surfaces of both hands and feet proximal to the metacarpophalangeal and metatarsophalangeal joints, respectively. Sensing electrodes were then placed on both sides of the body at the middle of an imaginary line on the dorsum of the wrist, joining the bony prominences of the radius and ulna, and at the middle of the anterior surface of the ankle, on an imaginary line joining the medial and lateral malleoli (Fig. 1A). The whole body (wrist-ankle model) was measured alternately on the left and right sides, and the average of the left and right measured values was used. Subsequently, the upper (wrist-ankle model) and lower limbs (ankle-ankle model) were measured in that order (Fig. 1B). Given that the electrical resistance of the component values varied with posture (21), the measurements were performed after >10 min with the participant in the supine position. The frequency of the BIS used in the present study ranged from 4 to 1 MHz. The resistance values of the extracellular and intracellular components (Re and Ri, respectively) were obtained from a Cole-Cole plot (22) using ImpediMed multifrequency analysis software (version 5.4.0.3). The settings of the analysis software were established based on previous studies (23,24). In the event that the BIS measurements deviated considerably from the norm, they were excluded from analysis due to the possibility of measurement error.

The lean body mass of the whole body was calculated from the resistance of the component values of the whole body using the formula installed as a standard in the analysis software. To calculate the impedance index (L^2^/Ri), the square of the sum of the left and right upper limb lengths and shoulder widths, and the square of the sum of the left and right lower limb lengths were divided by Ri (25). However, no estimation equations have been established to calculate body composition and water content of the upper and lower limbs in Japanese individuals. Instead, since L^2^/Ri in the upper and lower limbs reflects the respective muscle mass (26,27), the upper limb L^2^/Ri was designated as the ‘upper limb muscle mass index’ and the lower limb L^2^/Ri as the ‘lower limb muscle mass index’ (28). Additionally, the f characteristic was determined based on the energy required to supply a constant current through the tissue, reflecting the heterogeneity of the tissue density. The membrane capacitance, indicative of the retention capacity of the membrane potential gradient and depolarization reactivity of the muscle cell membranes, was also estimated (29,30). Finally, the phase angle of the whole body, upper limbs and lower limbs was estimated; the phase angle measurement for the whole body has been linked to adiposity and is considered to positively reflect the cell membrane content (9,12,29). Impedance (characteristic impedance; Zc) and reactance (characteristic resistance; Rc) at the characteristic frequency, f characteristic, and membrane capacitance were automatically calculated using analysis software. The phase angle was calculated using the following equation: Phase angle=[arc cosign (Rc/Zc) x (180/π)].

### Statistical analysis

The data for descriptive statistics are presented as the mean ± standard deviation. The Shapiro-Wilk test was used to analyze the distribution of each variable; variables with non-normal distributions were analyzed following log transformation. Continuous variables were analyzed using a one-way analysis of variance (ANOVA) and the Tukey-Kramer post hoc test in cases of significant differences. Qualitative variables were analyzed using Fisher's exact test. Cut-off values for determining the degree of grip strength by the variables of body composition or electrical characteristics were estimated using receiver operating characteristic (ROC) analysis. The effect size was also measured and reported using partial eta-squared (η_p_^2^) (0.01, 0.06 and 0.14 were considered to be small, medium and large effect sizes, respectively). Additionally, the effect size was calculated using Cohen's d (0.20, 0.50 and 0.80 reflected small, medium and large effect sizes, respectively) (31). The post hoc sample size (statistical power: 1-β) was calculated using the study data with the G-Power version 3.1.9.4 (University of Dusseldorf, Dusseldorf, Germany) to determine the statistical power of the study. A two-tailed P-value of P<0.05 was considered to indicate a statistically significant difference, unless otherwise stated.

## Results

Of the families of the 54 residents of the three group homes who received briefings explaining the study protocol, 21 families were excluded. More specifically, four families refused participation; 1 patient was hospitalized due to worsening conditions; 3 patients were bedridden for >1 week; 6 patients were male (removed due to the small number of males); for 6 patients, suitable BIS data could not be obtained due to the effects of metal bolts in the body; and patient could not perform BIS lower extremity measurements due to fractures in both legs. Finally, 33 patient-family sets were enrolled in the present study and included in the analyses.

The average age of the patients was 88.1±5.2 years; 57.6% of the patients had moderate or severe dementia (CDR1, 42.4%; CDR2, 21.2%; CDR3, 36.4%), and AD was the most common cause of dementia (66.7%; n=22; Table I). A total of 20 participants (60.6%) successfully achieved grip measurable strength measurements ≥5 kgf (high group), while 6 patients (18.2%) were non-measurable, being able to confirm grip strength, but were assigned a value of 0 kgf (<5 kgf; low group); the median value of all measurements between these two groups was 12.4 kgf. However, 7 patients (21.2%) were unable to perform the measurement and were therefore judged to be non-measurable by cognitive function. The BIS-estimated measurements of body composition, muscle mass and electrical properties were widely distributed (Table II).

No significant differences were detected in the rates of AD, nursing care level, or the history of diabetes, hypertension or dyslipidemia among the three groups categorized by grip strength. However, the high group had a significantly lower CDR and significantly higher BI scores than the low and non-measurable groups (Table III).

Although there were no abnormal values found in the BIS upper limb measurements, 9 patients had abnormal data in the lower limb measurements (5 patients had an artificial bone in their thigh, 2 patients had edema or inflammation in both legs, and 2 patients had suspected errors in measurements unrelated to these other causes). In whole-body BIS, there were no significant differences among the three groups in the parameters representing fat-free mass and electrical properties. However, as regards the upper limb, one-way ANOVA revealed significant differences in the characteristics of the three groups with large effect sizes (P=0.006, η_p_^2^=0.287). Subsequent post hoc analysis revealed that the f characteristic of the high group was significantly lower than that of the low (P=0.019, Cohen's d=1.184, 1-β=0.825) and non-measurable (P=0.016, Cohen's d=1.324, 1-β=0.761) groups. In addition, a significant difference in membrane capacitance was found among the three groups (P=0.005, η_p_^2^=0.298), with higher values in the high group compared with the low (P=0.020, Cohen's d=1.123, 1-β=0.785) and non-measurable (P=0.011, Cohen's d=1.573, 1-β=0.887) groups. Conversely, there was no significant difference in the phase angle of the upper limbs among the three groups nor in the lower limb values among the three groups (Table III). These results indicated that the high group had better f characteristics and membrane capacitance, which reflect the quality of upper limb muscles, compared to the low and non-measurable groups.

**Figure 2:**
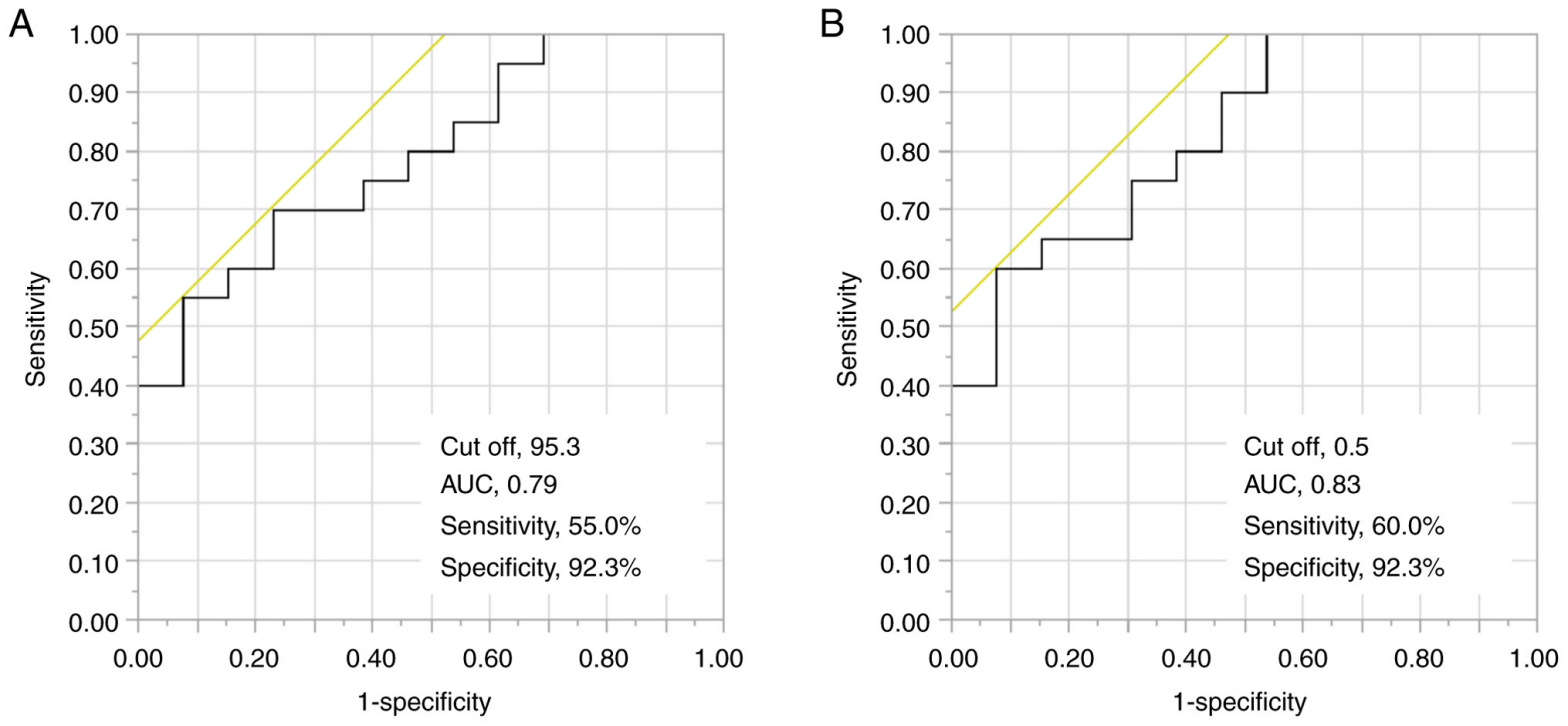
Receiver operating characteristic curve of the electrical properties used to predict grip strength: (A) f-characteristics; and (B) membrane capacitance.

Finally, the cut-off values for variables in which significant differences were detected in grip strength were calculated using ROC analysis. The cut-off value for f-characteristics was 95.3 (area under the ROC curve [AUC], 0.79; sensitivity, 55.0%; specificity, 92.3%), while that for membrane capacitance was 0.5 (AUC, 0.83; sensitivity, 60.0%; specificity 92.3%) (Fig. 2).

## Discussion

The present study detected a significant association between grip strength and the electronic properties of the upper limbs in females with advanced dementia aged 77-97 years and identified as needing care. To the best of our knowledge, the present study is the first to demonstrate that the f characteristics and membrane capacitance reflect grip strength values in patients with advanced dementia, indicating that they may represent novel indicators for the development, progression and prevention of severe sarcopenia in older patients with dementia. The number of participants in the present study was relatively small; however, the post hoc sample size (1-β) analysis revealed a power of ~80% (i.e., 76.1-88.7%), considering the minimum power for sufficient participants. Furthermore, the effect size (η_p_^2^ and Cohen's d) indicated significant differences between the groups. Therefore, it was deemed that the data obtained in the present study were reliable.

Previous studies have reported that muscle strength and muscle mass decline as dementia progresses; however, muscle strength declines before muscle mass (32). This may be explained by the condition of dynapenia (33,34), in which the limb skeletal muscle mass is maintained, but the muscle strength is weakened, or by weakened muscle quality (32). A decline in muscle quality has been observed in older adults (35), as well as in patients with dementia and diabetes (36,37); however, the detailed mechanisms involved are unclear.

In the present study, a significant association was observed between grip strength and electronic properties, particularly the f characteristics and membrane capacitance. This is consistent with a previous study that investigated the association among lower limb muscle strength, body composition and electrical properties in Japanese participants aged 26-76 years (29). However, differences between the upper and lower limbs were reported (29). Namely, electrical properties better reflect muscle function than body composition, supporting the possibility that electrical properties reflect muscle quality. It has been suggested that the phase angle is determined by the body-water ratio of muscle tissue and structural properties of muscle cells, as well as the f characteristics and membrane capacitance; it has also been shown to be significantly related to grip strength and motor function (11). In the present study, a significant association between grip strength, f-characteristics and membrane capacitance was observed only in the upper limbs. This suggests that grip strength, an indicator of upper arm muscle strength, is related to impedance values that reflect upper arm muscle mass. Given that grip strength is theoretically related to whole-body muscle strength, the use of cut-off values for muscle strength estimation obtained in the present study could be developed. This may serve as a novel strategy for the assessment of sarcopenia and muscle quality in patients with dementia for whom the estimation of muscle strength is difficult.

A previous study examining the association between lower limb muscle strength and phase angles identified a significant association between them; however, the correlation coefficient was lower than that of the f characteristic and membrane capacitance; thus, the phase angle was rejected as an explanatory variable for muscle strength by the stepwise method (29). Therefore, the phase angle may have a weaker association with muscle function than other measures of electrical properties, with no association between the two in the study population examined herein, which included patients for whom grip strength was not measurable due to cognitive decline. Notably, the standard deviations of the lower limbs were larger than those of the upper limbs due to large outliers. However, these outlier variables did not affect the main results of the study as they were appropriately log-transformed and statistically analyzed. Although details regarding the associated mechanism are unknown, these outlier patients reported experiencing pain in their lower limbs and inflammation in their thighs due to cellulitis. Hence, future studies are required to examine how electrical properties are affected by such inflammation.

Grip strength was also found to be significantly associated with CDR and BI, which may have been confounding factors owing to the association among grip strength, f characteristics and membrane capacitance; however, the details are unclear. Moreover, the CDR is an index of cognitive function, and BI is an index of ADL, both of which clearly differ from the f characteristics and membrane capacitance. Other potential confounding factors that were not measured in the present study, such as dietary protein intake (38), dietary diversity (39) and physical activity (40), may also affect grip strength. In the future, it will be necessary to re-examine a larger population to clarify their inter-relationships.

Several other limitations were also noted in the present study. First, there was a generalization limitation due to the small number of participants. However, the post hoc sample size analyses revealed the minimum power required for the sample size r, and the effect size demonstrated a meaningful magnitude of the differences between the groups. Second, the results of the present study were based exclusively on females; hence, the associations between muscle strength, muscle mass and electrical properties in older males with dementia remain unclear, limiting the external validity of the results. Studies are, therefore, required to examine whether sex-based differences exist in muscle quality, while considering potential confounding factors, such as diet and physical activity that were not investigated in the present study. Third, in the BIS measurement, it was impossible to standardize whether participants had eaten meals before the measurements were conducted or how long they had been in the supine position. Although the maximum resistance variation due to diet and postural changes is reportedly ~30 Ω in the whole body (41), the possibility that these factors affected the measurement results cannot be excluded. Fourth, in the measurement of grip strength, the measuring device could not measure grip strength values <5 kgf; therefore, the grip strength value of some participants was assumed to be 0 kgf and included in the data as such. However, the results of the study were the same even when the grip strength values of the participants were analyzed with values of 4 kgf instead of 0 kgf. In the future, more detailed data should be collected using a measurement device that can detect grip strength values <5 kgf. Despite these limitations, the present study carefully investigated the association between grip strength, body composition and electrical properties in older Japanese women identified as having Certification of Needed Long-Term Care with advanced dementia residing in group homes. The authors consider that that the findings presented herein may serve as an important resource for future studies on how to improve care for the physical functions of older individuals with dementia.

In conclusion, the present study demonstrates that a significant association exists between grip strength and the f characteristics and membrane capacitance, which are indicators of upper limb muscle quality measured using BIS, in older female residents identified as needing care in dementia group homes. The present study may provide critical theoretical data which can be used in the future to examine strategies with which to improve the physical function of residents with dementia in group homes.

## Figures and Tables

**Table I:** Basic characteristics of the study participants (n=33).

Characteristic	Value
Age, years; mean ± SD	88.1±5.2
Alzheimer's disease, n (%)	22 (66.7)
Nursing care level, n (%)	
1	6 (18.2)
2	7 (21.2)
3	13 (39.3)
4	4 (12.1)
5	3 (9.1)
Clinical dementia rating, n (%)	
1	14 (42.4)
2	7 (21.2)
3	12 (36.4)
Diabetes, n (%)	2 (6.1)
Hypertension, n (%)	7 (21.2)
Hyperlipidemia, n (%)	4 (12.1)
Height (cm), mean ± SD	144.3±5.6
Weight (kg), mean ± SD	44.2±7.9
Body mass index (kg/m^2^), mean ± SD	21.3±4.0

SD, standard deviation.

**Table II:** Body composition, muscle mass and electrical characteristics measured using the BIS and grip strength values of the study participants.

Parameter	Value
Whole body	
Re (Ω)	673±113
Ri (Ω)	2,700±1,323
ECW (L)	10.5±1.6
ICW (L)	12.8±3.2
TBW (L)	23.3±4.3
FFM (kg)	31.8±5.9
% Fat mass (%)	27.4±9.9
f characteristic (kHz)	91.7±35.4
Membrane capacitance (nF)	0.7±0.4
Phase angle (deg)	4.3±2.2
Upper limbs	
Re (Ω)	761±104
Ri (Ω)	3,025±1,081
Upper limbs muscle mass index (cm^2^/Ω)	6.1±2.5
f characteristic (kHz)	102.4±63.2
Membrane capacitance (nF)	0.5±0.2
Phase angle (deg)	3.7±0.8
Lower limbs^a^	
Re (Ω)	545±140
Ri (Ω)	2,868±1,079
Lower limbs muscle mass index (cm^2^/Ω)	7.4±2.4
f characteristic (kHz)	75.0±23.3
Membrane capacitance (nF)	0.8±0.3
Phase angle (deg)	3.1±0.7
Grip strength	
Measurable, ≥5 kgf	20 (60.6)
Non-measurable, <5 kgf	6 (18.2)
Non-measurable by cognitive function	7 (21.2)
Grip strength, measurable (kgf)	14.2±4.4
Grip strength, measurable/weight (kgf/kg)	0.3±0.1

Data are expressed as n (%) or the mean ± standard deviation.

^a^A total of 9 participants were excluded as their BIS measurements deviated significantly, due to the risk of measurement error (n=24). BIS, bioimpedance spectroscopy; Re, extracellular resistance; Ri, intracellular resistance; ECW, extracellular water; ICW, intracellular water; TBW, total body water; FMM, fat-free mass; f characteristic, characteristic frequency.

**Table III:** Association between grip strength and body composition and electrical properties estimated using BIS values.

		Grip strength			Post hoc test			
Parameter	High group (n=13)	Low group (n=13)	Non-measurable group (n=7)	P-value	High vs. low group	High vs. non-measurable group	Low vs. non-measurable group	Effect size
Age (years), mean ± SD	88.3±5.5	88.3±4.6	87.4±6.1	0.933				0.005
Alzheimer disease (yes/no)	7/6	8/5	7/0	0.096				
Nursing care level (1/2/3/4/5)	5/4/4/0/0	0/0/4/1/2	1/3/5/3/1	0.060				
Clinical dementia rating (1/2/3)	8/4/1	5/3/5	1/0/6	0.016				
Diabetes (yes/no)	2/11	0/13	0/7	0.335				
Hypertension (yes/no)	3/10	1/12	3/4	0.227				
Hyperlipidemia (yes/no)	3/10	1/12	0/7	0.236				
Barthel index (score), mean ± SD	68.1±15.1^a^	46.2±27.2^b^	19.3±19.2^c^	0.001	0.038	<0.001	0.021	0.459
Height (cm), mean ± SD	146.2±5.3	142.5±5.4	144.1±6.1	0.251				0.088
Weight (kg), mean ± SD	46.9±6.7	41.4±8.3	44.3±8.2	0.212				0.098
Body mass index (kg/m^2^), mean ± SD	22.0±3.4	20.5±4.6	21.4±4.3	0.546				0.040
Whole body, mean ± SD								
ECW (L)	11.5±1.4^a^	9.9±1.5^b^	9.9±1.5^b^	0.010	0.016	0.045	0.999	0.264
ICW (L)	13.7±2.4	12.3±3.8	11.9±3.6	0.271				0.083
TBW (L)	25.2±3.5	22.2±4.4	21.8±5.0	0.121				0.131
FFM (kg)	34.4±4.7	30.4±5.9	29.8±6.8	0.121				0.131
% Fat mass (%)	26.2±7.5	25.9±10.2	32.2±12.9	0.380				0.062
f characteristic (kHz)	80.2±25.8	105.0±47.5	88.3±12.0	0.090				0.149
Membrane capacitance (nF)	0.8±0.3	0.6±0.2	0.7±0.5	0.149				0.119
Phase angle (deg)	4.0±0.8	4.5±3.0	4.5±2.5	0.942				0.004
Upper limbs, mean ± SD								
Upper limbs muscle mass index (cm^2^/Ω)	6.5±1.4	5.3±1.6	6.9±4.5	0.233				0.093
f characteristic (kHz)	73.2±15.1^a^	110.7±52.8^b^	141.3±107.3^b^	0.006	0.019	0.016	0.845	0.287
Membrane capacitance (nF)	0.7±0.2^a^	0.5±0.2^b^	0.4±0.2^b^	0.005	0.020	0.011	0.745	0.298
Phase angle (deg)	3.9±0.5	3.5±1.0	3.8±1.0	0.577				0.036
Lower limbs^d^, mean ± SD								
Lower limbs muscle mass index (cm^2^/Ω)	8.2±2.2	7.0±2.9	6.4±1.9	0.323				0.102
f characteristic (kHz)	69.5±10.4	77.5±35.9	82.1±24.4	0.552				0.055
Membrane capacitance (nF)	0.9±0.3	0.7±0.4	0.6±0.3	0.106				0.192
Phase angle (deg)	3.2±0.4	2.9±0.8	3.2±0.8	0.700				0.033

Data were analyzed using Fisher's exact test or one way-ANOVA following logarithmic transformation.

^a^,

^b^,

^c^Significant differences between groups (P<0.05; Tukey's post hoc test). Effect size was calculated by partial eta-squared (η_p_^2^): 0.01, 0.06 and 0.14 were considered as small, medium and large effect sizes, respectively. High group, measurable and higher than the median value in terms grip strength; low group, measurable and lower than the median value in terms of grip strength; non-measurable group, not measurable according to cognitive function.

^d^A total of 9 participants were excluded as their BIS measurements deviated significantly, due to the risk of measurement error (n=24: n=11 for the high group, n=7 for the low group, and n=6 for the non-measurable group). BIS, bioimpedance spectroscopy; SD, standard deviation; Re, extracellular resistance; Ri, intracellular resistance; ECW, extracellular water; ICW, intracellular water; TBW, total body water; FFM, fat-free mass; f characteristic, characteristic frequency.

## Data Availability

The datasets used and/or analyzed during the current study are available from the corresponding author on reasonable request.
